# A rare case report of reversible acute kidney injury due to hyperuricemia alone

**DOI:** 10.1007/s11255-020-02743-0

**Published:** 2021-01-03

**Authors:** Yan Zhang, Wei-Xiu Wang, Xiao-Xiao Zhang, Man-Yu Zhang, Ya-Ru Ren, Ding-Wei Yang

**Affiliations:** 1 Division of Nephrology, Department of Internal Medicine, Tianjin Hospital, No. 406 Jiefang South Road, Hexi, Tianjin, 300211 China; 2 Graduate School, Tianjin Medical University, Tianjin, China

Editor,

Acute uric acid nephropathy is one of the causes of acute kidney injury (AKI), which usually occurs in patients with tumor lysis syndrome and postoperative cardiopulmonary [1, 2]. AKI due to hyperuricemia alone is rare. Here, we reported a patient who developed AKI due to elevated serum uric acid (UA) solely and recovered completely after treatment.

A 27-year-old male with a history of gout diagnosed 2 years ago was admitted for nausea, vomit and serum creatinine (Scr) increasing for 1 day with no significant past history and medical history. His eating habits were profound intake of purine-rich foods. Physical examination showed: temperature was 37.4 °C, blood pressure was 140/93 mmHg, ankle edema, and no other abnormalities. Investigations revealed extremely elevated UA, Scr and blood urea nitrogen (BUN), which increased to 1123, 676 and 23.54, respectively, from reference values (reference range were 208–428 μmol/L, 61.9–114.9 μmol/L and 3.1–8.0 mmol/L, respectively) and decreased UA in the urine of 1187.5 (reference range was 2400–5400 μmol/24 h). The urine UA-to-creatinine ratio was 1.76 and a large number of uric acid crystals were found in the urinary sediment, which was consistent with acute uric acid nephropathy [3]. Serum cystatin C and serum β_2_ microglobulin increased mildly. Hepatitis B virus (HBV) surface antigen, HBV e-antigen and HBV core antibody were positive in the serum. Hemodialysis was used to remove metabolic wastes. Febuxostat, methylprednisolone and tenofovir alafenamide fumarate were also added. One day after treatment, levels of serum UA, Scr and BUN quickly corrected to 671 μmol/L, 537 μmol/L and 17.26 mmol/L significantly with clinical symptoms resolved. Maintenance therapy with hemodialysis was used again on day 3. After 5 days’ treatment, levels of serum UA, Scr and BUN were decreased to 461 μmol/L, 163 μmol/L and 9.76 mmol/L, respectively, with sustained urine volume being 1500–2500 mL. On day 7, he was discharged with the levels of Scr and BUN normal (96 μmol/L and 7.8 mmol/L, respectively) and serum UA being 440 μmol/L.

AKI can be caused by hyperuricemia alone. A high-purine diet, increased purine metabolism and excessive alcohol consumption could result in increasing UA production rapidly [4]. Uric acid crystal-induced tubular obstruction is associated with AKI [5, 6]. Oxidative stress, activation of renin–angiotensin system and inflammasome pathway also contribute to AKI [5–7]. Hyperuricemia-induced AKI always presents with elevated serum UA levels (typically > 10–15 mg/dL), presence of uric acid crystals in the renal sediment, and an elevated urine UA-to-creatinine ratio of > 1 [3]. The pathologic features of acute uric acid nephropathy are reversible. Renal function is expected to be completely restored if hemodialysis is initiated in time to rapidly reduce serum UA and Scr. Febuxostat and corticosteroids are considered to be efficacious in serum UA lowering and protecting the renal function [8–10].

In conclusion, this was a unique case of AKI caused by extremely high increase in serum UA level alone. The pathologic features of hyperuricemia-induced AKI are reversible. With timely recognition and management, the renal function can be restored completely.
